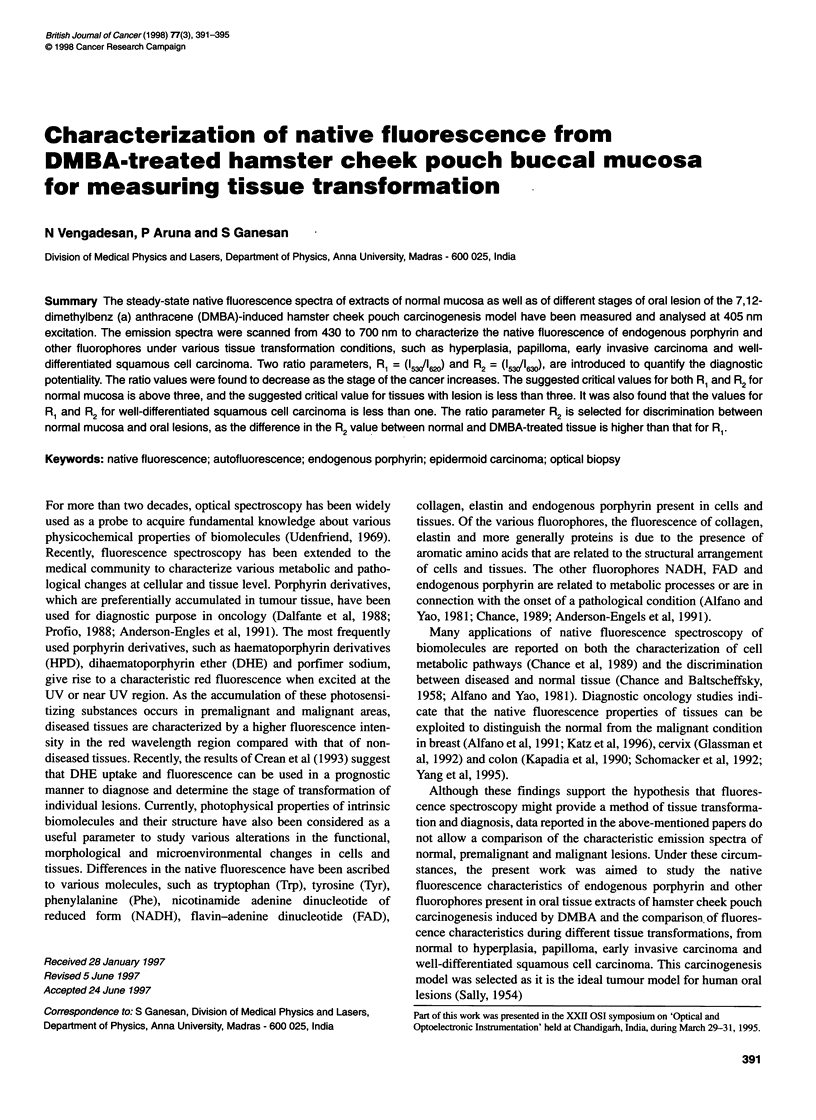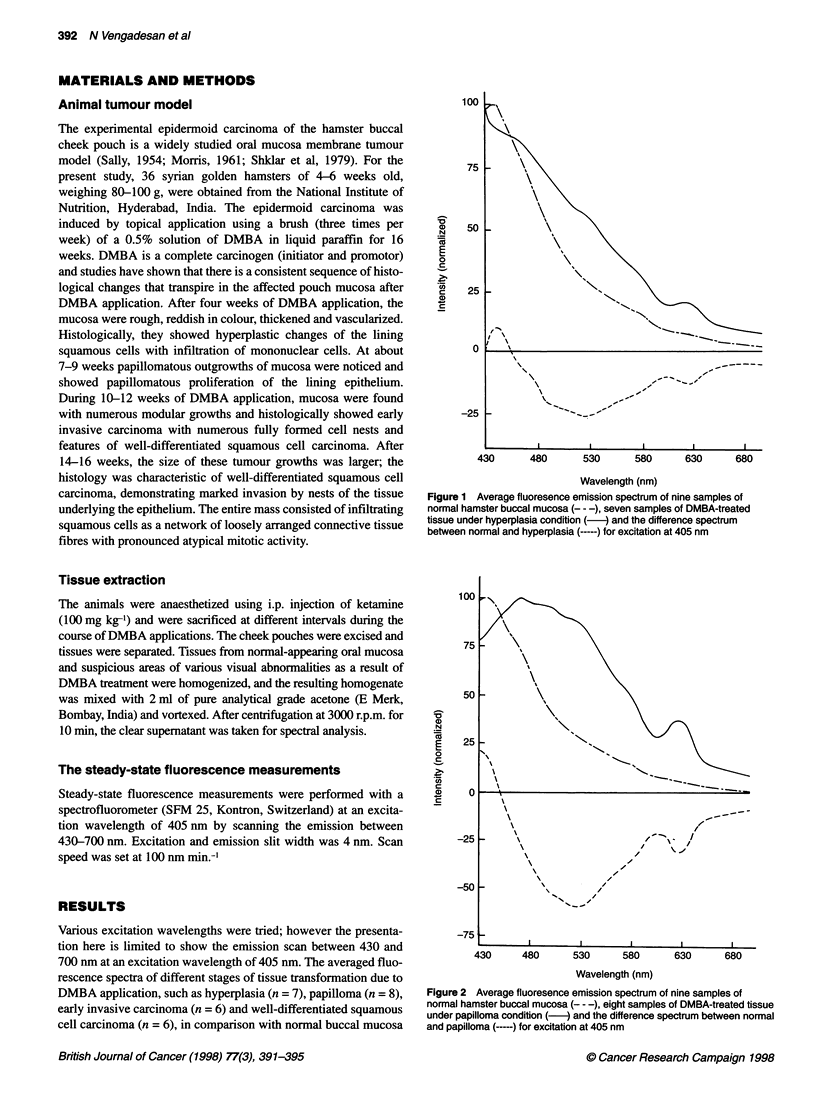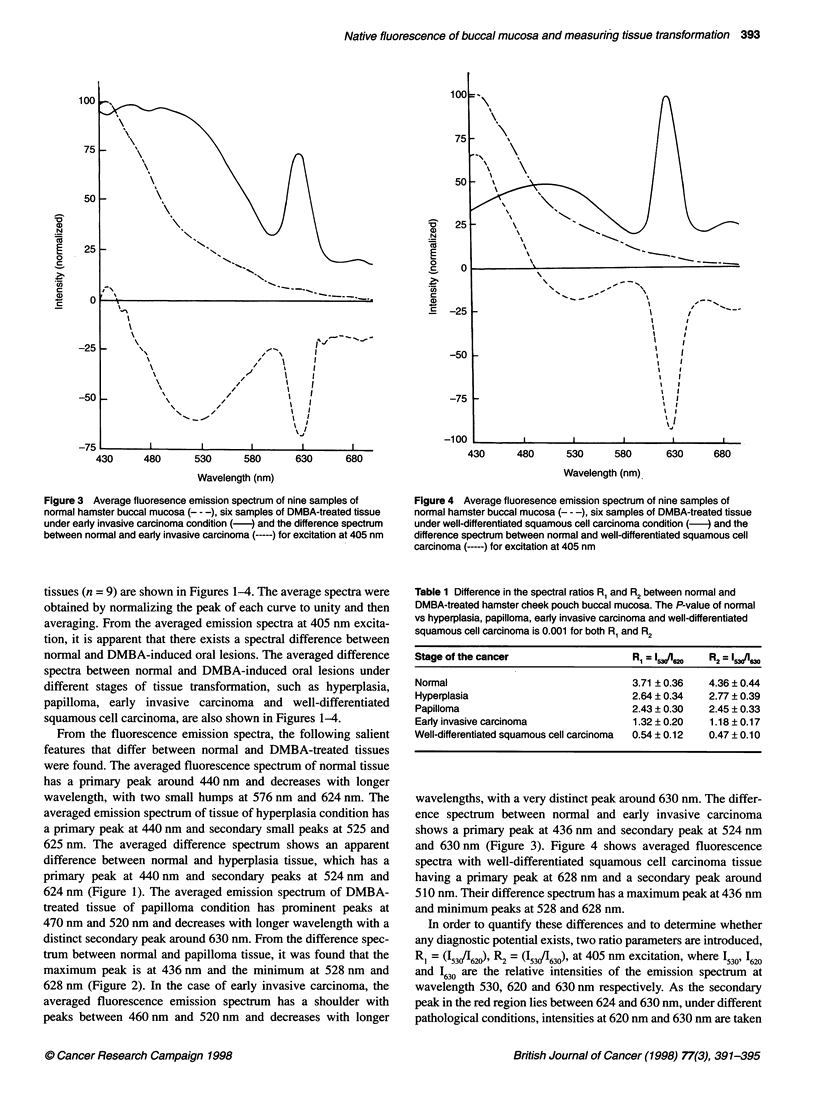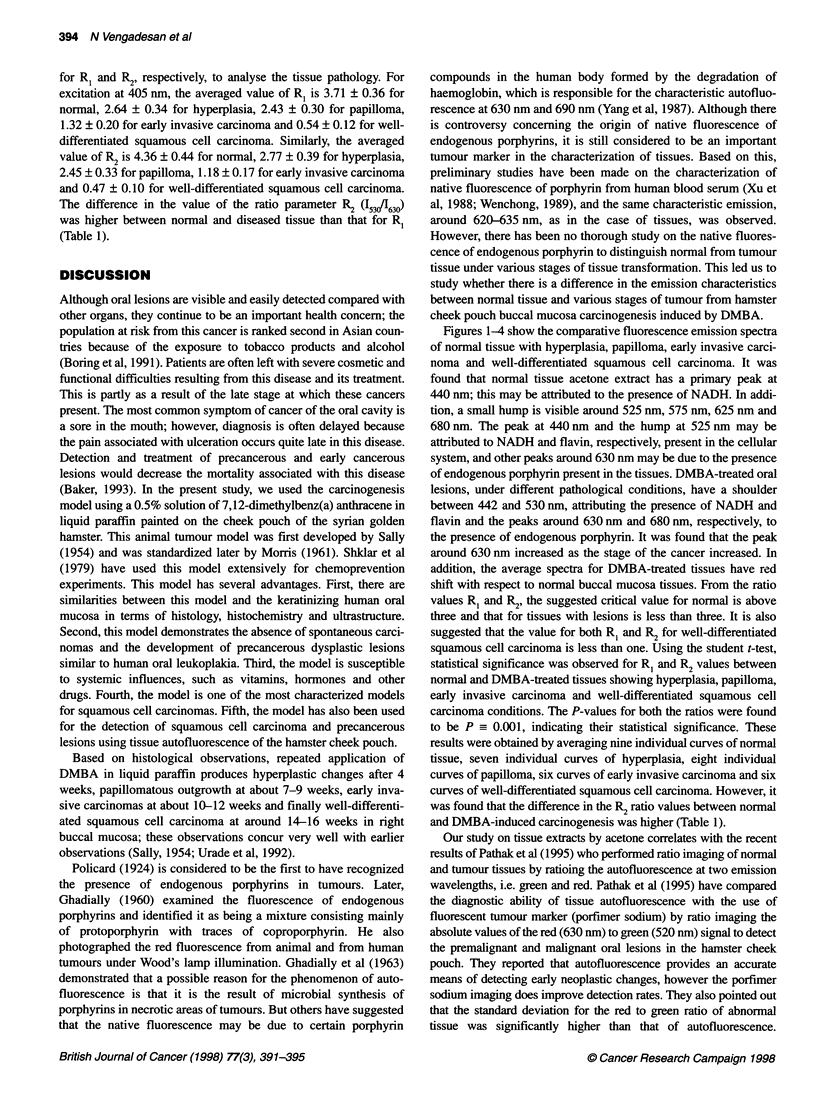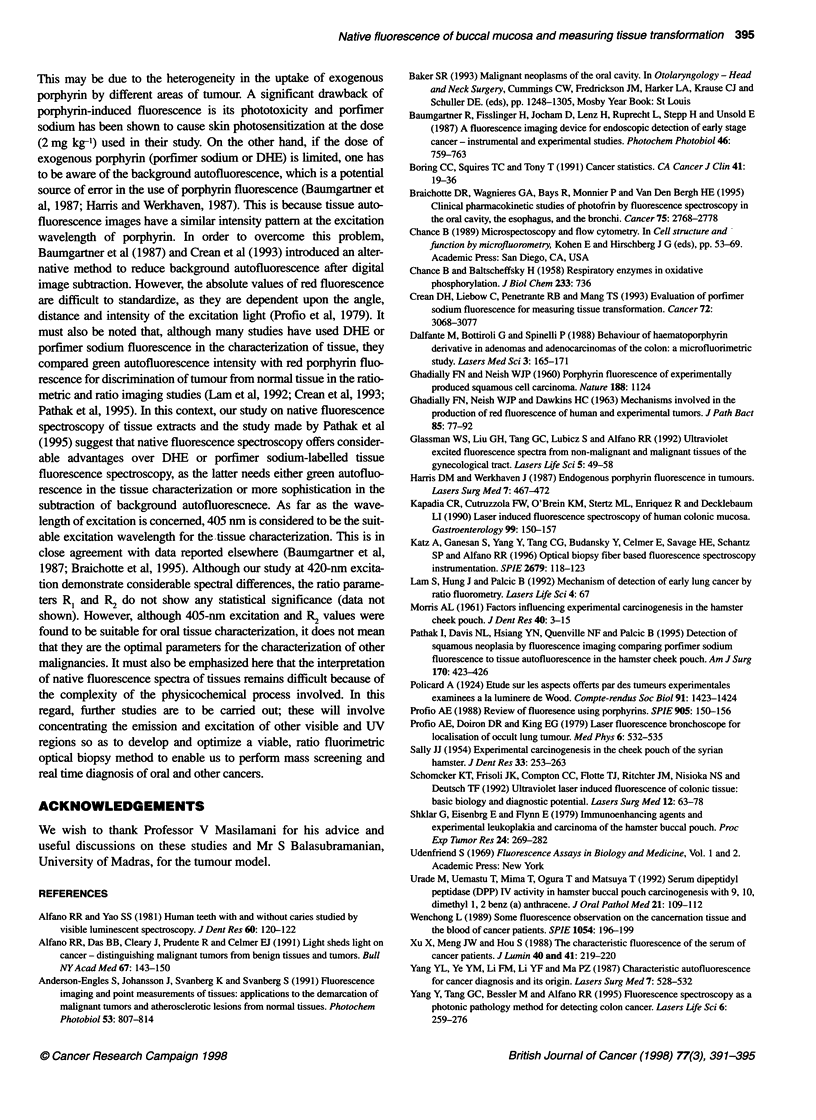# Characterization of native fluorescence from DMBA-treated hamster cheek pouch buccal mucosa for measuring tissue transformation.

**DOI:** 10.1038/bjc.1998.62

**Published:** 1998

**Authors:** N. Vengadesan, P. Aruna, S. Ganesan

**Affiliations:** Department of Physics, Anna University, Madras, India.

## Abstract

The steady-state native fluorescence spectra of extracts of normal mucosa as well as of different stages of oral lesion of the 7,12-dimethylbenz (a) anthracene (DMBA)-induced hamster cheek pouch carcinogenesis model have been measured and analysed at 405 nm excitation. The emission spectra were scanned from 430 to 700 nm to characterize the native fluorescence of endogenous porphyrin and other fluorophores under various tissue transformation conditions, such as hyperplasia, papilloma, early invasive carcinoma and well-differentiated squamous cell carcinoma. Two ratio parameters, R1 = (I530/I620) and R2 = (I530/I630), are introduced to quantify the diagnostic potentiality. The ratio values were found to decrease as the stage of the cancer increases. The suggested critical values for both R1 and R2 for normal mucosa is above three, and the suggested critical value for tissues with lesion is less than three. It was also found that the values for R1 and R2 for well-differentiated squamous cell carcinoma is less than one. The ratio parameter R2 is selected for discrimination between normal mucosa and oral lesions, as the difference in the R2 value between normal and DMBA-treated tissue is higher than that for R1.


British Journal of Cancer (1998) 77(3), 391–395
© 1998 Cancer Research Campaign

### Characterization of native fluorescence from DMBA-treated hamster cheek pouch buccal mucosa for measuring tissue transformation

**N Vengadesan, P Aruna and S Ganesan**

Division of Medical Physics and Lasers, Department of Physics, Anna University, Madras - 600 025, India

**Summary** The steady-state native fluorescence spectra of extracts of normal mucosa as well as of different stages of oral lesion of the 7,12-dimethylbenz (a) anthracene (DMBA)-induced hamster cheek pouch carcinogenesis model have been measured and analysed at 405 nm excitation. The emission spectra were scanned from 430 to 700 nm to characterize the native fluorescence of endogenous porphyrin and other fluorophores under various tissue transformation conditions, such as hyperplasia, papilloma, early invasive carcinoma and well-differentiated squamous cell carcinoma. Two ratio parameters, $R_1 = (I_{530}/I_{620})$ and $R_2 = (I_{530}/I_{630})$, are introduced to quantify the diagnostic potentiality. The ratio values were found to decrease as the stage of the cancer increases. The suggested critical values for both $R_1$ and $R_2$ for normal mucosa is above three, and the suggested critical value for tissues with lesion is less than three. It was also found that the values for $R_1$ and $R_2$ for well-differentiated squamous cell carcinoma is less than one. The ratio parameter $R_2$ is selected for discrimination between normal mucosa and oral lesions, as the difference in the $R_2$ value between normal and DMBA-treated tissue is higher than that for $R_1$.

**Keywords:** native fluorescence; autofluorescence; endogenous porphyrin; epidermoid carcinoma; optical biopsy

For more than two decades, optical spectroscopy has been widely used as a probe to acquire fundamental knowledge about various physicochemical properties of biomolecules (Udenfriend, 1969). Recently, fluorescence spectroscopy has been extended to the medical community to characterize various metabolic and pathological changes at cellular and tissue level. Porphyrin derivatives, which are preferentially accumulated in tumour tissue, have been used for diagnostic purpose in oncology (Dalfante et al, 1988; Profio, 1988; Anderson-Engles et al, 1991). The most frequently used porphyrin derivatives, such as haematoporphyrin derivatives (HPD), dihaematoporphyrin ether (DHE) and porfimer sodium, give rise to a characteristic red fluorescence when excited at the UV or near UV region. As the accumulation of these photosensitizing substances occurs in premalignant and malignant areas, diseased tissues are characterized by a higher fluorescence intensity in the red wavelength region compared with that of non-diseased tissues. Recently, the results of Crean et al (1993) suggest that DHE uptake and fluorescence can be used in a prognostic manner to diagnose and determine the stage of transformation of individual lesions. Currently, photophysical properties of intrinsic biomolecules and their structure have also been considered as a useful parameter to study various alterations in the functional, morphological and microenvironmental changes in cells and tissues. Differences in the native fluorescence have been ascribed to various molecules, such as tryptophan (Trp), tyrosine (Tyr), phenylalanine (Phe), nicotinamide adenine dinucleotide of reduced form (NADH), flavin–adenine dinucleotide (FAD), collagen, elastin and endogenous porphyrin present in cells and tissues. Of the various fluorophores, the fluorescence of collagen, elastin and more generally proteins is due to the presence of aromatic amino acids that are related to the structural arrangement of cells and tissues. The other fluorophores NADH, FAD and endogenous porphyrin are related to metabolic processes or are in connection with the onset of a pathological condition (Alfano and Yao, 1981; Chance, 1989; Anderson-Engels et al, 1991).

Many applications of native fluorescence spectroscopy of biomolecules are reported on both the characterization of cell metabolic pathways (Chance et al, 1989) and the discrimination between diseased and normal tissue (Chance and Baltscheffsky, 1958; Alfano and Yao, 1981). Diagnostic oncology studies indicate that the native fluorescence properties of tissues can be exploited to distinguish the normal from the malignant condition in breast (Alfano et al, 1991; Katz et al, 1996), cervix (Glassman et al, 1992) and colon (Kapadia et al, 1990; Schomacker et al, 1992; Yang et al, 1995).

Although these findings support the hypothesis that fluorescence spectroscopy might provide a method of tissue transformation and diagnosis, data reported in the above-mentioned papers do not allow a comparison of the characteristic emission spectra of normal, premalignant and malignant lesions. Under these circumstances, the present work was aimed to study the native fluorescence characteristics of endogenous porphyrin and other fluorophores present in oral tissue extracts of hamster cheek pouch carcinogenesis induced by DMBA and the comparison of fluorescence characteristics during different tissue transformations, from normal to hyperplasia, papilloma, early invasive carcinoma and well-differentiated squamous cell carcinoma. This carcinogenesis model was selected as it is the ideal tumour model for human oral lesions (Sally, 1954)

*Received 28 January 1997*
*Revised 5 June 1997*
*Accepted 24 June 1997*

*Correspondence to:* S Ganesan, Division of Medical Physics and Lasers, Department of Physics, Anna University, Madras - 600 025, India

Part of this work was presented in the XXII OSI symposium on 'Optical and Optoelectronic Instrumentation' held at Chandigarh, India, during March 29–31, 1995.

### MATERIALS AND METHODS

### Animal tumour model

The experimental epidermoid carcinoma of the hamster buccal cheek pouch is a widely studied oral mucosa membrane tumour model (Sally, 1954; Morris, 1961; Shklar et al, 1979). For the present study, 36 syrian golden hamsters of 4–6 weeks old, weighing 80–100 g, were obtained from the National Institute of Nutrition, Hyderabad, India. The epidermoid carcinoma was induced by topical application using a brush (three times per week) of a 0.5% solution of DMBA in liquid paraffin for 16 weeks. DMBA is a complete carcinogen (initiator and promotor) and studies have shown that there is a consistent sequence of histological changes that transpire in the affected pouch mucosa after DMBA application. After four weeks of DMBA application, the mucosa were rough, reddish in colour, thickened and vascularized. Histologically, they showed hyperplastic changes of the lining squamous cells with infiltration of mononuclear cells. At about 7–9 weeks papillomatous outgrowths of mucosa were noticed and showed papillomatous proliferation of the lining epithelium. During 10–12 weeks of DMBA application, mucosa were found with numerous modular growths and histologically showed early invasive carcinoma with numerous fully formed cell nests and features of well-differentiated squamous cell carcinoma. After 14–16 weeks, the size of these tumour growths was larger; the histology was characteristic of well-differentiated squamous cell carcinoma, demonstrating marked invasion by nests of the tissue underlying the epithelium. The entire mass consisted of infiltrating squamous cells as a network of loosely arranged connective tissue fibres with pronounced atypical mitotic activity.

**Figure 1** Average fluoresence emission spectrum of nine samples of normal hamster buccal mucosa (– – –), seven samples of DMBA-treated tissue under hyperplasia condition (——) and the difference spectrum between normal and hyperplasia (-----) for excitation at 405 nm

### Tissue extraction

The animals were anaesthetized using i.p. injection of ketamine (100 mg $kg^{-1}$) and were sacrificed at different intervals during the course of DMBA applications. The cheek pouches were excised and tissues were separated. Tissues from normal-appearing oral mucosa and suspicious areas of various visual abnormalities as a result of DMBA treatment were homogenized, and the resulting homogenate was mixed with 2 ml of pure analytical grade acetone (E Merk, Bombay, India) and vortexed. After centrifugation at 3000 r.p.m. for 10 min, the clear supernatant was taken for spectral analysis.

### The steady-state fluorescence measurements

Steady-state fluorescence measurements were performed with a spectrofluorometer (SFM 25, Kontron, Switzerland) at an excitation wavelength of 405 nm by scanning the emission between 430–700 nm. Excitation and emission slit width was 4 nm. Scan speed was set at 100 nm $min.^{-1}$

### RESULTS

Various excitation wavelengths were tried; however the presentation here is limited to show the emission scan between 430 and 700 nm at an excitation wavelength of 405 nm. The averaged fluorescence spectra of different stages of tissue transformation due to DMBA application, such as hyperplasia ($n$ = 7), papilloma ($n$ = 8), early invasive carcinoma ($n$ = 6) and well-differentiated squamous cell carcinoma ($n$ = 6), in comparison with normal buccal mucosa

**Figure 2** Average fluoresence emission spectrum of nine samples of normal hamster buccal mucosa (– – –), eight samples of DMBA-treated tissue under papilloma condition (——) and the difference spectrum between normal and papilloma (-----) for excitation at 405 nm

© Cancer Research Campaign 1998

**Figure 3** Average fluoresence emission spectrum of nine samples of normal hamster buccal mucosa (– - –), six samples of DMBA-treated tissue under early invasive carcinoma condition (——) and the difference spectrum between normal and early invasive carcinoma (-----) for excitation at 405 nm

**Figure 4** Average fluoresence emission spectrum of nine samples of normal hamster buccal mucosa (– - –), six samples of DMBA-treated tissue under well-differentiated squamous cell carcinoma condition (——) and the difference spectrum between normal and well-differentiated squamous cell carcinoma (-----) for excitation at 405 nm tissues ($n = 9$) are shown in Figures 1–4. The average spectra were obtained by normalizing the peak of each curve to unity and then averaging. From the averaged emission spectra at 405 nm excitation, it is apparent that there exists a spectral difference between normal and DMBA-induced oral lesions. The averaged difference spectra between normal and DMBA-induced oral lesions under different stages of tissue transformation, such as hyperplasia, papilloma, early invasive carcinoma and well-differentiated squamous cell carcinoma, are also shown in Figures 1–4.

From the fluorescence emission spectra, the following salient features that differ between normal and DMBA-treated tissues were found. The averaged fluorescence spectrum of normal tissue has a primary peak around 440 nm and decreases with longer wavelength, with two small humps at 576 nm and 624 nm. The averaged emission spectrum of tissue of hyperplasia condition has a primary peak at 440 nm and secondary small peaks at 525 and 625 nm. The averaged difference spectrum shows an apparent difference between normal and hyperplasia tissue, which has a primary peak at 440 nm and secondary peaks at 524 nm and 624 nm (Figure 1). The averaged emission spectrum of DMBA-treated tissue of papilloma condition has prominent peaks at 470 nm and 520 nm and decreases with longer wavelength with a distinct secondary peak around 630 nm. From the difference spectrum between normal and papilloma tissue, it was found that the maximum peak is at 436 nm and the minimum at 528 nm and 628 nm (Figure 2). In the case of early invasive carcinoma, the averaged fluorescence emission spectrum has a shoulder with peaks between 460 nm and 520 nm and decreases with longer wavelengths, with a very distinct peak around 630 nm. The difference spectrum between normal and early invasive carcinoma shows a primary peak at 436 nm and secondary peak at 524 nm and 630 nm (Figure 3). Figure 4 shows averaged fluorescence spectra with well-differentiated squamous cell carcinoma tissue having a primary peak at 628 nm and a secondary peak around 510 nm. Their difference spectrum has a maximum peak at 436 nm and minimum peaks at 528 and 628 nm.

**Table 1** Difference in the spectral ratios $R_1$ and $R_2$ between normal and DMBA-treated hamster cheek pouch buccal mucosa. The *P*-value of normal vs hyperplasia, papilloma, early invasive carcinoma and well-differentiated squamous cell carcinoma is 0.001 for both $R_1$ and $R_2$

| Stage of the cancer | $R_1 = I_{530}/I_{620}$ | $R_2 = I_{530}/I_{630}$ |
|---|---|---|
| Normal | 3.71 ± 0.36 | 4.36 ± 0.44 |
| Hyperplasia | 2.64 ± 0.34 | 2.77 ± 0.39 |
| Papilloma | 2.43 ± 0.30 | 2.45 ± 0.33 |
| Early invasive carcinoma | 1.32 ± 0.20 | 1.18 ± 0.17 |
| Well-differentiated squamous cell carcinoma | 0.54 ± 0.12 | 0.47 ± 0.10 |

In order to quantify these differences and to determine whether any diagnostic potential exists, two ratio parameters are introduced, $R_1 = (I_{530}/I_{620})$, $R_2 = (I_{530}/I_{630})$, at 405 nm excitation, where $I_{530}$, $I_{620}$ and $I_{630}$ are the relative intensities of the emission spectrum at wavelength 530, 620 and 630 nm respectively. As the secondary peak in the red region lies between 624 and 630 nm, under different pathological conditions, intensities at 620 nm and 630 nm are taken

© Cancer Research Campaign 1998 

for $R_1$ and $R_2$, respectively, to analyse the tissue pathology. For excitation at 405 nm, the averaged value of $R_1$ is 3.71 ± 0.36 for normal, 2.64 ± 0.34 for hyperplasia, 2.43 ± 0.30 for papilloma, 1.32 ± 0.20 for early invasive carcinoma and 0.54 ± 0.12 for well-differentiated squamous cell carcinoma. Similarly, the averaged value of $R_2$ is 4.36 ± 0.44 for normal, 2.77 ± 0.39 for hyperplasia, 2.45 ± 0.33 for papilloma, 1.18 ± 0.17 for early invasive carcinoma and 0.47 ± 0.10 for well-differentiated squamous cell carcinoma. The difference in the value of the ratio parameter $R_2$ ($I_{530}/I_{630}$) was higher between normal and diseased tissue than that for $R_1$ (Table 1).

### DISCUSSION

Although oral lesions are visible and easily detected compared with other organs, they continue to be an important health concern; the population at risk from this cancer is ranked second in Asian countries because of the exposure to tobacco products and alcohol (Boring et al, 1991). Patients are often left with severe cosmetic and functional difficulties resulting from this disease and its treatment. This is partly as a result of the late stage at which these cancers present. The most common symptom of cancer of the oral cavity is a sore in the mouth; however, diagnosis is often delayed because the pain associated with ulceration occurs quite late in this disease. Detection and treatment of precancerous and early cancerous lesions would decrease the mortality associated with this disease (Baker, 1993). In the present study, we used the carcinogenesis model using a 0.5% solution of 7,12-dimethylbenz(a) anthracene in liquid paraffin painted on the cheek pouch of the syrian golden hamster. This animal tumour model was first developed by Sally (1954) and was standardized later by Morris (1961). Shklar et al (1979) have used this model extensively for chemoprevention experiments. This model has several advantages. First, there are similarities between this model and the keratinizing human oral mucosa in terms of histology, histochemistry and ultrastructure. Second, this model demonstrates the absence of spontaneous carcinomas and the development of precancerous dysplastic lesions similar to human oral leukoplakia. Third, the model is susceptible to systemic influences, such as vitamins, hormones and other drugs. Fourth, the model is one of the most characterized models for squamous cell carcinomas. Fifth, the model has also been used for the detection of squamous cell carcinoma and precancerous lesions using tissue autofluorescence of the hamster cheek pouch.

Based on histological observations, repeated application of DMBA in liquid paraffin produces hyperplastic changes after 4 weeks, papillomatous outgrowth at about 7–9 weeks, early invasive carcinomas at about 10–12 weeks and finally well-differentiated squamous cell carcinoma at around 14–16 weeks in right buccal mucosa; these observations concur very well with earlier observations (Sally, 1954; Urade et al, 1992).

Policard (1924) is considered to be the first to have recognized the presence of endogenous porphyrins in tumours. Later, Ghadially (1960) examined the fluorescence of endogenous porphyrins and identified it as being a mixture consisting mainly of protoporphyrin with traces of coproporphyrin. He also photographed the red fluorescence from animal and from human tumours under Wood's lamp illumination. Ghadially et al (1963) demonstrated that a possible reason for the phenomenon of autofluorescence is that it is the result of microbial synthesis of porphyrins in necrotic areas of tumours. But others have suggested that the native fluorescence may be due to certain porphyrin compounds in the human body formed by the degradation of haemoglobin, which is responsible for the characteristic autofluorescence at 630 nm and 690 nm (Yang et al, 1987). Although there is controversy concerning the origin of native fluorescence of endogenous porphyrins, it is still considered to be an important tumour marker in the characterization of tissues. Based on this, preliminary studies have been made on the characterization of native fluorescence of porphyrin from human blood serum (Xu et al, 1988; Wenchong, 1989), and the same characteristic emission, around 620–635 nm, as in the case of tissues, was observed. However, there has been no thorough study on the native fluorescence of endogenous porphyrin to distinguish normal from tumour tissue under various stages of tissue transformation. This led us to study whether there is a difference in the emission characteristics between normal tissue and various stages of tumour from hamster cheek pouch buccal mucosa carcinogenesis induced by DMBA.

Figures 1–4 show the comparative fluorescence emission spectra of normal tissue with hyperplasia, papilloma, early invasive carcinoma and well-differentiated squamous cell carcinoma. It was found that normal tissue acetone extract has a primary peak at 440 nm; this may be attributed to the presence of NADH. In addition, a small hump is visible around 525 nm, 575 nm, 625 nm and 680 nm. The peak at 440 nm and the hump at 525 nm may be attributed to NADH and flavin, respectively, present in the cellular system, and other peaks around 630 nm may be due to the presence of endogenous porphyrin present in the tissues. DMBA-treated oral lesions, under different pathological conditions, have a shoulder between 442 and 530 nm, attributing the presence of NADH and flavin and the peaks around 630 nm and 680 nm, respectively, to the presence of endogenous porphyrin. It was found that the peak around 630 nm increased as the stage of the cancer increased. In addition, the average spectra for DMBA-treated tissues have red shift with respect to normal buccal mucosa tissues. From the ratio values $R_1$ and $R_2$, the suggested critical value for normal is above three and that for tissues with lesions is less than three. It is also suggested that the value for both $R_1$ and $R_2$ for well-differentiated squamous cell carcinoma is less than one. Using the student *t*-test, statistical significance was observed for $R_1$ and $R_2$ values between normal and DMBA-treated tissues showing hyperplasia, papilloma, early invasive carcinoma and well-differentiated squamous cell carcinoma conditions. The *P*-values for both the ratios were found to be $P \equiv 0.001$, indicating their statistical significance. These results were obtained by averaging nine individual curves of normal tissue, seven individual curves of hyperplasia, eight individual curves of papilloma, six curves of early invasive carcinoma and six curves of well-differentiated squamous cell carcinoma. However, it was found that the difference in the $R_2$ ratio values between normal and DMBA-induced carcinogenesis was higher (Table 1).

Our study on tissue extracts by acetone correlates with the recent results of Pathak et al (1995) who performed ratio imaging of normal and tumour tissues by ratioing the autofluorescence at two emission wavelengths, i.e. green and red. Pathak et al (1995) have compared the diagnostic ability of tissue autofluorescence with the use of fluorescent tumour marker (porfimer sodium) by ratio imaging the absolute values of the red (630 nm) to green (520 nm) signal to detect the premalignant and malignant oral lesions in the hamster cheek pouch. They reported that autofluorescence provides an accurate means of detecting early neoplastic changes, however the porfimer sodium imaging does improve detection rates. They also pointed out that the standard deviation for the red to green ratio of abnormal tissue was significantly higher than that of autofluorescence.

© Cancer Research Campaign 1998

This may be due to the heterogeneity in the uptake of exogenous porphyrin by different areas of tumour. A significant drawback of porphyrin-induced fluorescence is its phototoxicity and porfimer sodium has been shown to cause skin photosensitization at the dose (2 mg $kg^{-1}$) used in their study. On the other hand, if the dose of exogenous porphyrin (porfimer sodium or DHE) is limited, one has to be aware of the background autofluorescence, which is a potential source of error in the use of porphyrin fluorescence (Baumgartner et al, 1987; Harris and Werkhaven, 1987). This is because tissue autofluorescence images have a similar intensity pattern at the excitation wavelength of porphyrin. In order to overcome this problem, Baumgartner et al (1987) and Crean et al (1993) introduced an alternative method to reduce background autofluorescence after digital image subtraction. However, the absolute values of red fluorescence are difficult to standardize, as they are dependent upon the angle, distance and intensity of the excitation light (Profio et al, 1979). It must also be noted that, although many studies have used DHE or porfimer sodium fluorescence in the characterization of tissue, they compared green autofluorescence intensity with red porphyrin fluorescence for discrimination of tumour from normal tissue in the ratiometric and ratio imaging studies (Lam et al, 1992; Crean et al, 1993; Pathak et al, 1995). In this context, our study on native fluorescence spectroscopy of tissue extracts and the study made by Pathak et al (1995) suggest that native fluorescence spectroscopy offers considerable advantages over DHE or porfimer sodium-labelled tissue fluorescence spectroscopy, as the latter needs either green autofluorescence in the tissue characterization or more sophistication in the subtraction of background autofluorescnece. As far as the wavelength of excitation is concerned, 405 nm is considered to be the suitable excitation wavelength for the tissue characterization. This is in close agreement with data reported elsewhere (Baumgartner et al, 1987; Braichotte et al, 1995). Although our study at 420-nm excitation demonstrate considerable spectral differences, the ratio parameters $R_1$ and $R_2$ do not show any statistical significance (data not shown). However, although 405-nm excitation and $R_2$ values were found to be suitable for oral tissue characterization, it does not mean that they are the optimal parameters for the characterization of other malignancies. It must also be emphasized here that the interpretation of native fluorescence spectra of tissues remains difficult because of the complexity of the physicochemical process involved. In this regard, further studies are to be carried out; these will involve concentrating the emission and excitation of other visible and UV regions so as to develop and optimize a viable, ratio fluorimetric optical biopsy method to enable us to perform mass screening and real time diagnosis of oral and other cancers.

### ACKNOWLEDGEMENTS

We wish to thank Professor V Masilamani for his advice and useful discussions on these studies and Mr S Balasubramanian, University of Madras, for the tumour model.

### REFERENCES

Alfano RR and Yao SS (1981) Human teeth with and without caries studied by visible luminescent spectroscopy. *J Dent Res* **60**: 120–122

Alfano RR, Das BB, Cleary J, Prudente R and Celmer EJ (1991) Light sheds light on cancer – distinguishing malignant tumors from benign tissues and tumors. *Bull NY Acad Med* **67**: 143–150

Anderson-Engles S, Johansson J, Svanberg K and Svanberg S (1991) Fluorescence imaging and point measurements of tissues: applications to the demarcation of malignant tumors and atherosclerotic lesions from normal tissues. *Photochem Photobiol* **53**: 807–814

Baker SR (1993) Malignant neoplasms of the oral cavity. In *Otolaryngology – Head and Neck Surgery*, Cummings CW, Fredrickson JM, Harker LA, Krause CJ and Schuller DE. (eds), pp. 1248–1305, Mosby Year Book: St Louis

Baumgartner R, Fisslinger H, Jocham D, Lenz H, Ruprecht L, Stepp H and Unsold E (1987) A fluorescence imaging device for endoscopic detection of early stage cancer – instrumental and experimental studies. *Photochem Photobiol* **46**: 759–763

Boring CC, Squires TC and Tony T (1991) Cancer statistics. *CA Cancer J Clin* **41**: 19–36

Braichotte DR, Wagnieres GA, Bays R, Monnier P and Van Den Bergh HE (1995) Clinical pharmacokinetic studies of photofrin by fluorescence spectroscopy in the oral cavity, the esophagus, and the bronchi. *Cancer* **75**: 2768–2778

Chance B (1989) Microspectoscopy and flow cytometry. In *Cell structure and function by microfluorometry*, Kohen E and Hirschberg J G (eds), pp. 53–69. Academic Press: San Diego, CA, USA

Chance B and Baltscheffsky H (1958) Respiratory enzymes in oxidative phosphorylation. *J Biol Chem* **233**: 736

Crean DH, Liebow C, Penetrante RB and Mang TS (1993) Evaluation of porfimer sodium fluorescence for measuring tissue transformation. *Cancer* **72**: 3068–3077

Dalfante M, Bottiroli G and Spinelli P (1988) Behaviour of haematoporphyrin derivative in adenomas and adenocarcinomas of the colon: a microfluorimetric study. *Lasers Med Sci* **3**: 165–171

Ghadially FN and Neish WJP (1960) Porphyrin fluorescence of experimentally produced squamous cell carcinoma. *Nature* **188**: 1124

Ghadially FN, Neish WJP and Dawkins HC (1963) Mechanisms involved in the production of red fluorescence of human and experimental tumors. *J Path Bact* **85**: 77–92

Glassman WS, Liu GH, Tang GC, Lubicz S and Alfano RR (1992) Ultraviolet excited fluorescence spectra from non-malignant and malignant tissues of the gynecological tract. *Lasers Life Sci* **5**: 49–58

Harris DM and Werkhaven J (1987) Endogenous porphyrin fluorescence in tumours. *Lasers Surg Med* **7**: 467–472

Kapadia CR, Cutruzzola FW, O'Brein KM, Stertz ML, Enriquez R and Decklebaum LI (1990) Laser induced fluorescence spectroscopy of human colonic mucosa. *Gastroenterology* **99**: 150–157

Katz A, Ganesan S, Yang Y, Tang CG, Budansky Y, Celmer E, Savage HE, Schantz SP and Alfano RR (1996) Optical biopsy fiber based fluorescence spectroscopy instrumentation. *SPIE* **2679**: 118–123

Lam S, Hung J and Palcic B (1992) Mechanism of detection of early lung cancer by ratio fluorometry. *Lasers Life Sci* **4**: 67

Morris AL (1961) Factors influencing experimental carcinogenesis in the hamster cheek pouch. *J Dent Res* **40**: 3–15

Pathak I, Davis NL, Hsiang YN, Quenville NF and Palcic B (1995) Detection of squamous neoplasia by fluorescence imaging comparing porfimer sodium fluorescence to tissue autofluorescence in the hamster cheek pouch. *Am J Surg* **170**: 423–426

Policard A (1924) Etude sur les aspects offerts par des tumeurs experimentales examinees a la luminere de Wood. *Compte-rendus Soc Biol* **91**: 1423–1424

Profio AE (1988) Review of fluoresence using porphyrins. *SPIE* **905**: 150–156

Profio AE, Doiron DR and King EG (1979) Laser fluorescence bronchoscope for localisation of occult lung tumour. *Med Phys* **6**: 532–535

Sally JJ (1954) Experimental carcinogenesis in the cheek pouch of the syrian hamster. *J Dent Res* **33**: 253–263

Schomcker KT, Frisoli JK, Compton CC, Flotte TJ, Ritchter JM, Nisioka NS and Deutsch TF (1992) Ultraviolet laser induced fluorescence of colonic tissue: basic biology and diagnostic potential. *Lasers Surg Med* **12**: 63–78

Shklar G, Eisenbrg E and Flynn E (1979) Immunoenhancing agents and experimental leukoplakia and carcinoma of the hamster buccal pouch. *Proc Exp Tumor Res* **24**: 269–282

Udenfriend S (1969) *Fluorescence Assays in Biology and Medicine*, Vol. 1 and 2. Academic Press: New York

Urade M, Uemastu T, Mima T, Ogura T and Matsuya T (1992) Serum dipeptidyl peptidase (DPP) IV activity in hamster buccal pouch carcinogenesis with 9, 10, dimethyl 1, 2 benz (a) anthracene. *J Oral Pathol Med* **21**: 109–112

Wenchong L (1989) Some fluorescence observation on the cancernation tissue and the blood of cancer patients. *SPIE* **1054**: 196–199

Xu X, Meng JW and Hou S (1988) The characteristic fluorescence of the serum of cancer patients. *J Lumin* **40 and 41**: 219–220

Yang YL, Ye YM, Li FM, Li YF and Ma PZ (1987) Characteristic autofluorescence for cancer diagnosis and its origin. *Lasers Surg Med* **7**: 528–532

Yang Y, Tang GC, Bessler M and Alfano RR (1995) Fluorescence spectroscopy as a photonic pathology method for detecting colon cancer. *Lasers Life Sci* **6**: 259–276

© Cancer Research Campaign 1998